# An empirical Bayes normalization method for connectivity metrics in resting state fMRI

**DOI:** 10.3389/fnins.2015.00316

**Published:** 2015-09-16

**Authors:** Shuo Chen, Jian Kang, Guoqing Wang

**Affiliations:** ^1^Department of Epidemiology and Biostatistics, University of MarylandCollege Park, MD, USA; ^2^Department of Biostatistics, University of MichiganAnn Arbor, MI, USA

**Keywords:** anticorrelation, connectivity, fMRI, network, normalization, resting state

## Abstract

Functional connectivity analysis using resting-state functional magnetic resonance imaging (rs-fMRI) has emerged as a powerful technique for investigating functional brain networks. The functional connectivity is often quantified by statistical metrics (e.g., Pearson correlation coefficient), which may be affected by many image acquisition and preprocessing steps such as the head motion correction and the global signal regression. The appropriate quantification of the connectivity metrics is essential for meaningful and reproducible scientific findings. We propose a novel empirical Bayes method to normalize the functional brain connectivity metrics on a posterior probability scale. Moreover, the normalization function maps the original connectivity metrics to values between zero and one, which is well-suited for the graph theory based network analysis and avoids the information loss due to the (negative value) hard thresholding step. We apply the normalization method to a simulation study and the simulation results show that our normalization method effectively improves the robustness and reliability of the quantification of brain functional connectivity and provides more powerful group difference (biomarkers) detection. We illustrate our method on an analysis of a rs-fMRI dataset from the Autism Brain Imaging Data Exchange (ABIDE) study.

## 1. Introduction

Resting-state fMRI (rs-fMRI) has been applied to study functional brain connectivity patterns and networks in the absence of external stimuli (Biswal et al., 1995; Beckmann et al., 2005; Fransson, 2005; De Luca et al., 2006; Fox et al., 2006). Many previous rs-fMRI studies have identified altered functional connectivity expressions and networks from different clinical populations (Dosenbach et al., 2007; Greicius, 2008; Fornito et al., 2012). To investigate the properties of the complex brain functional connectivity networks, the graph theory models have been developed and yielded many meaningful findings (Braun et al., 2009; Bullmore and Sporns, 2009; Rubinov and Sporns, 2010).

The functional connectivity analyses are often conducted based on connectivity metrics rather than the raw time courses from rs-fMRI data. There have been many functional connectivity metrics employed to measure the functional coherence of temporal profiles between two distinct brain areas, for example, Pearson correlation coefficients, mutual information coefficients, and spectral coherence (Zhou et al., 2009; Smith, 2012). Therefore, the functional connectivity strength is often quantified by a calculated statistic (most times a scalar), and hence the reproducibility and validity of the following group level statistical inferences are heavily impacted by the statistical quantification method and choice of connectivity metric. However, the connectivity metrics could be sensitive to the changes of image acquisition and preprocessing procedures. For example, in the debate of whether global trend regression should be applied, it has been pointed out that such preprocessing step may shift the whole connectivity distribution (using the Pearson correlation coefficient metric) toward -1 and introduce false anticorrelations (Fox et al., 2009; Murphy et al., 2009; Weissenbacher et al., 2009; Chai et al., 2012). It brings up the practical trade-off between specificity of anticorrelation and the alignment of the scales of correlation value distributions across subjects. Although the agreement (of whether global signal regression should be used) has not been reached, it is clear that the scaling of the connectivity metrics can be influenced by many (preprocessing) factors and substantial noises (Murphy et al., 2013).

The brain functional connectivity often aims to identify the differentially expressed connections between brain areas for different cohorts. To provide valid and reproducible group level functional connectivity inferences for these studies, we are ought to assign proper values to the input connectivity metrics which are proportional to the true connectivity strength and comparable across subjects. Thus, the appropriate scaling and rescaling methods toward the raw connectivity metrics of the high-dimensional connectivity expressions are desired, which is often referred as a “normalization” step. The feature expression normalization has been widely used as a key standard preprocessing step for most of the high-throughput “omics” data, (e.g., the quantile normalization for gene expression microarray data) in order to mitigate the subjectwise systematic shift/noises and to improve the accuracy of differential expression detection by transforming the expression metrics to a comparable scale across subjects (Bolstad et al., 2003; Bullard et al., 2010; Robinson and Oshlack, 2010; Hansen et al., 2012). The normalization plays a crucial role in group level analysis of high-throughput data since the sensitivity, specificity, and reproducibility of differential expression detection rely on the proper quantification of the expression metrics. However, the normalization step has been rarely applied to brain functional connectomics data, though similar subject-wise systematic shift/noises may also exist in functional connectivity data. The appropriate normalization method is expected to be robust to the measurement shifts/noises and to provide a comparable connectivity expression metric across subjects. In addition, when studying the complex functional brain connectivity network, we often employ the graph theoretical models which require the scale of connectivity expression ranging between zero and one (“Binarization”) (Rubinov and Sporns, 2010; Smith, 2012). When the Pearson correlation coefficient is used, the correlation values below zero are often (hard) thresholded (“Thresholding”) (Rubinov and Sporns, 2010; Smith, 2012). However, thresholding or binarization of the continuous connectivity expression values could lead to substantial information loss(Harrell, 2001). Thus, a normalization approach which maps connectivity metrics to the support between zero and one is also desired.

To address the above unmet needs of functional connectivity analysis, we present a new empirical Bayes normalization method for rs-fMRI connectivity analysis. The method has three main advantages: (1) it mitigates subjectwise systematic shift/noises and provides robust normalized metrics to ensure the connectivity metrics comparable between subjects; (2) the normalized metrics improve differential expression detection for true biomarker detection; (3) it quantifies the connectivity expression value ranging between zero and one which is well-suited for graph theoretical models. In this article, we use Pearson correlation for demonstration because it is most widely used and studied (Zalesky et al., 2012), though the proposed normalization method can be applied to any functional connectivity metrics.

## 2. Methods

In this section, we illustrate the normalization method based on functional connectivity expressions between 90 nodes, which represents the commonly used first 90 Anatomical Automatic Labeling (AAL) regions in brain connectivity studies (Tzourio-Mazoyer et al., 2002; Zalesky et al., 2010b).

### 2.1. Distribution of connectivity

**Figure 1:**
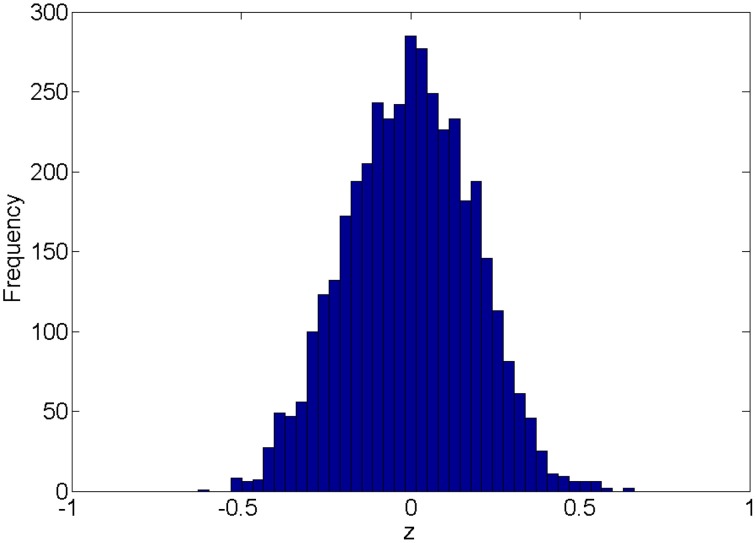
**Empirical distribution of pairwise correlations between 90 random white noise vectors**.

We first introduce the null distribution of 4005 pairwise correlations between time courses from 90 nodes. Each time course is a randomly simulated white noise vector (including 50 data points) with mean 0, and variance 1 and all time courses are generated independently. The resulting connectivity metric distribution is shown in Figure 1. The correlations range between (-0.63, 0.66) and are centered around 0. The 4005 sampling correlations (the calculated statistics) are used to quantify the connectivity expressions, and among those correlations there are many values close to 1 or -1 that are often considered as “false positively” correlated or anticorrelated. We denote the distribution in Figure 1 as the null distribution.

### 2.2. Normalization function

In practice, the distribution of connectivity expressions from rs-fMRI data is often mixed by the null distribution as well as the distributions from the “true positive” correlated or anticorrelated components (a small hump close to 1 or −1). Thus, there are more than one component in the distribution of correlations (i.e., a mixture distribution). Moreover, at the group-level the modes or medians of the correlation distributions from different subjects may shift apart significantly from each other, which may be a result of systematic measurement errors (e.g., in the image acquisition and preprocessing steps). The systematic shifts could cause connectivity expression metrics not comparable across subjects and then lead to a failure of group level inferences such as biomarker detection. To address such concerns, we propose a normalization method to quantify the connectivity expression when adjusting the probability of “false positives” and systematic shifts across subjects.

We denote the connectivity expression value by *z*, and the probability distribution by *f*(*z*). The connectivity expressions are high-throughput, lending itself to recognition of the pattern of “false positives” through a mixture model:

(2.1) $$\begin{array}{l} f\left(z\right)=p_{0}f_{0}\left(z\right)+p_{1}f_{1}\left(z\right), \end{array}$$

where *p*_0_ = *Pr*{*uncorrelated*(*null*)} and *f*_0_(*z*) is the probability density distribution (pdf) for the null component; and *p*_1_ = *Pr*{*correlated*(*non*−*null*)} and *f*_1_(*z*) is the pdf for the non-null component. The mixture distribution is defined identically to the local false discovery rate model (*fdr*) proposed by Efron (2004). *f*_0_(*z*) and *f*_1_(*z*) are either parametric distributions such as normal distributions or non/semi-parametric (empirical distributions) (Wu et al., 2006; Strimmer, 2008). However, different from the interest of detecting the local false positive rate of the statistic *z*, our goal is to assign a normalized value *g*(*z*) to each connectivity expression metric *z* (*g* is the mapping/normalization function).

In the mixture model, we can estimate the probability of *z* from the non-null component and the null component. Given *p*_0_, *f*_0_(*z*), *p*_1_, *f*_1_(*z*), the posterior probability of a connectivity belonging to the non-null component at *z* is

(2.2) $$\begin{array}{l} g\left(z\right)=p_{1}f_{1}\left(z\right)∕f\left(z\right)=1-p_{0}f_{0}\left(z\right)∕f\left(z\right), \end{array}$$

which equals to one minus local false discovery rate fdr(z). We use *g*(*z*) as the normalization function of the connectivity metric *z*, which represents the estimated posterior probability of *z* being truly connected or anticorrelated.

The normalization function *g*(*z*) generally yields a higher probability value when *z* is larger, but rather than a linear relationship it depends on the parameters and distributions of {*p*_0_, *f*_0_(*z*), *p*_1_, *f*_1_(*z*)}. However, in practice, the prior parameters and distributions {*p*_0_, *f*_0_(*z*), *p*_1_, *f*_1_(*z*)} are unknown and are often estimated from the observed data of *z* parametricly or nonparametricly.

Our normalization method is called an empirical Bayes method because the normalized connectivity expression is the posterior probability of *z* from *f*_1_, and the model parameters {*p*_0_, *f*_0_(*z*), *p*_1_, *f*_1_(*z*)} are estimated directly rather by sampling from the full conditionals. Fortunately, the estimation techniques for such type of empirical Bayes mixture model have been well-developed and thoroughly discussed (Efron, 2004; Wu et al., 2006; Strimmer, 2008; Schwartzman et al., 2009). For derivation and discussion of the detailed estimation procedure, we refer the readers to the original papers. Provided with the estimated $\left\{\hat{p_{0}},\hat{f_{0}\left(z\right)},\hat{p_{1}},\hat{f_{1}\left(z\right)}\right\}$, the estimated normalization function becomes

(2.3) $$\begin{array}{l} g_{s}\left(z\right)=\hat{p_{1}}\hat{f_{1}\left(z\right)}∕\left(\hat{p_{0}}\hat{f_{0}\left(z\right)}+\hat{p_{1}}\hat{f_{1}\left(z\right)}\right). \end{array}$$

The normalization function *g*_*s*_(*z*) is estimated based on a single subject/image *s* (*s* = 1, …, *N*, and *N* is the total number of subjects), as it is determined by the distribution of connectivity expression metrics of each individual. The normalized connectivity expressions are comparable across subjects because they are probability metrics. In general, only high-throughput expression data can include sufficient data points to obtain reliable prior parameter and distribution estimates ($\left\{\hat{p_{0}},\hat{f_{0}\left(z\right)},\hat{p_{1}},\hat{f_{1}\left(z\right)}\right\}$), hence we would apply the normalization method only when the pairwise connectivity metrics are calculated from at least 70 ROIs. The normalization procedure is conducted prior to the group level statistical inferences such as statistical tests and regressions to ensure the connectivity expression metrics being appropriately scaled and comparable across subjects. The statistical inferences based on normalized connectivity expression metrics could be less affected by the systematic shifts and random measurement errors, and hence are expected to be more robust and reproducible. We will demonstrate the properties of the normalization function in the simulation and data example sections. As the direct assessment of the normalization effect on connectivity metrics (calculated statistics) could be challenging, we examine the normalization method by comparing the statistical inferences based on normalized connectivity metrics and raw (non-normalized) connectivity metrics.

## 3. Simulations

In this section, we simulate a case-control rs-fMRI study to examine the performance of our normalization method. We generate 30 subjects for each group and within each subject we simulate 4005 correlation coefficients between 90 nodes/regions. We assume that the correlations between the first 30 ROIs are differentially expressed (the control group exhibits higher connections than the case group).

**Figure 2:**
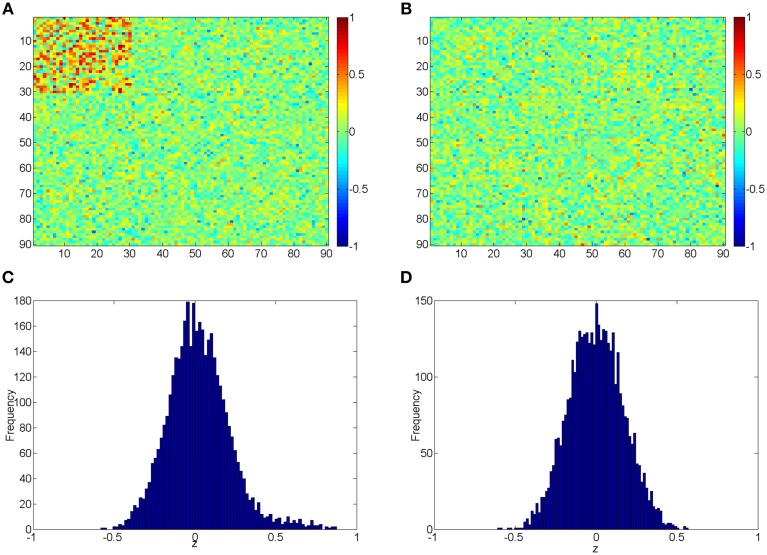
**(A,B)** Heatmaps of the simulated correlations of the control and case groups; **(C,D)** Histograms of the simulated correlations of the control and case groups.

The beta distribution is employed to simulate correlation coefficients because it is more flexible and better resembles the real distribution of correlation coefficients from rs-fMRI data than other distributions (e.g., Gaussian distribution) (Ji et al., 2005; Jantschi and Sorana, 2011). We generate *z*_1_ from the non-null distribution by a transformed Beta distribution: *x*_1_ ~ *Beta* (α_1_ = 3, β_1_ = 3) and *z*_1_ = 1.55*x*_1_ − 0.55 for correlation coefficients with higher connectivity expression levels; and *z*_0_ from the null distribution by *x*_0_ ~ *Beta* (α_0_ = 18, β_0_ = 18) and then *z*_0_ = *x*_0_ ∗ 2 − 1. *z*_1_ represent 435 highly expressed correlation coefficients between the first 30 nodes for each subject in the control group, and *z*_0_ represents the rest of correlations for subjects in control group and all correlations for subjects in the case group. In this way, all simulated correlations range from [−1, 1], and Figure 2 demonstrates the simulated data for case and control group. Additionally, we use different set of parameters to represent various patterns of correlation distribution (e.g., Murphy et al., 2009) including: (i) more dispersed null component *x*_0_ ~ *Beta* (α_0_ = 9, β_0_ = 9) (P_1_); (ii) right skewed connected component *x*_1_ ~ *Beta* (α_1_ = 2, β_1_ = 3) (P_2_); (iii) left skewed connected component *x*_1_ ~ *Beta* (α_1_ = 3, β_1_ = 2) (P_3_).

In addition, we simulate another scenario by adding systematic shifts across subjects by:

$$\begin{array}{ll} \mu_{s}\sim uniform\left(-0.2,0.2\right),\; & \;\\ {\tilde{z}}_{s}=z_{s}+N\left(\mu_{s},\sigma^{2}\right),\; & \; \end{array}$$

where ${\tilde{z}}_{s}$ represent the correlations for subject *s* with systematic shift and the values over -1 or 1 are set to -1 and 1 (**Figure 4A**). We use σ^2^ to indicate the magnitude of the shifts.

**Figure 3:**
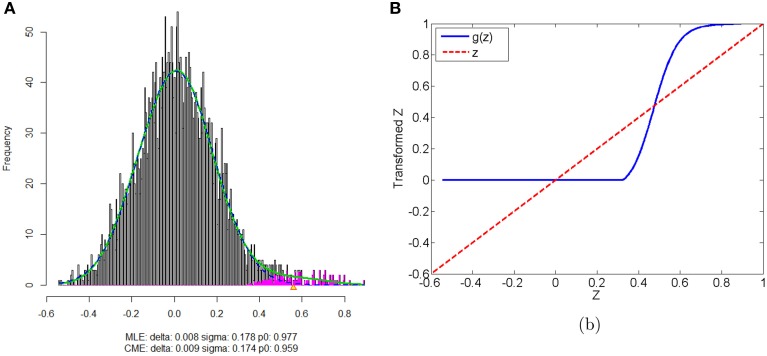
**(A)** Mixture model estimation procedure using “*locfdr*” in R; **(B)** Normalization function vs. Original correlations: the blue line is the normalization function that maps the raw connectivity metrics to normalized metrics; the red line is used a reference representing no normalization is applied.

We apply our normalization method to the simulated correlations with the main goal of differentially expressed connectivity discovery. The R (http://CRAN.R-project.org/) package “*locfdr*” is used to estimate the mixture model; and the normalization function *g*_*s*_(*z*) is calculated for each individual (see Example in Supplementary Material). Figure 3 shows that the mixture model is well estimated as well as the shape of the normalization function. Comparing to the original correlation or the variance stablizing transformation methods (e.g., Fisher's Z, probit, or logit transformed correlations), the the posterior probability based normalization function incorporates the “false positive” belief with observed connectivity expressions by empirical Bayes framework. The normalized correlations ares not related to the original correlations linearly, but monotonely increasing. *g*(*z*) increases steeply between around 0.4 and 0.6 because the posterior belief of “true positive” rises drastically. If there are both “true positive” correlation and anticorrelation components, then three components will be detected and estimated and two normalization functions are provided separately for positive and negative correlations (see details in Section 5).

**Figure 4:**
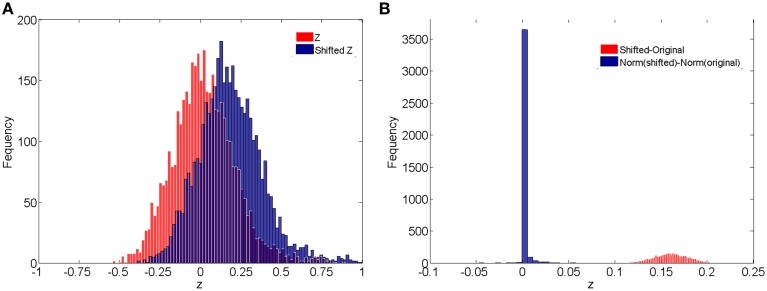
**(A)** Histograms of correlations and correlations with systematic shifts for a subject in the control group; **(B)** Histograms of the change of the shift.

In addition, we compare the raw correlations (without and with subject systematic shifts) with the normalized connectivity expressions to investigate the effects of normalization in connectivity metric quantification and differentially expressed connectivity detection. Different levels of subject systematic shifts (different σ^2^) are also included. We first evaluate the effects of normalization on random shifts. If there is a random shift from the random measurement error, Figure 4A demonstrates the histograms of the original correlations (red) and systematically (with randomness) shifted correlations (blue) for a subject in the control group. Figure 4B illustrates the impact of the systematic shifts on the non-normalized and the normalized connectivity expression. The red histogram in Figure 4B shows the difference of original correlations and shifted correlations. Thus, if there is a systematic shift the connectivity will be affected with consistent bias, which may cause invalid group level inferences. The blue histogram in Figure 4B shows the differences of normalized original correlations and normalized shifted correlations which are distributed around 0. Clearly, the normalized connectivity metric is almost invariant to the systematic shifts, therefore the normalization algorithm improves the robustness of the connectivity metrics to systematic shifts/noises.

We then examine the performance of our normalization method on differential expression detection (the main aim). We conduct the two sample Wilcoxon signed-rank non-parametric tests (α = 0.05) on the 4005 connectivity metrics *z* and normalized connectivity *g*(*z*) under both non-shifted and shifted scenarios. As we evaluate simultaneous multiple tests, the FDR (with *q* = 0.1 as the threshold) is applied to adjust multiple testing in the simulation study.

**Figure 5:**
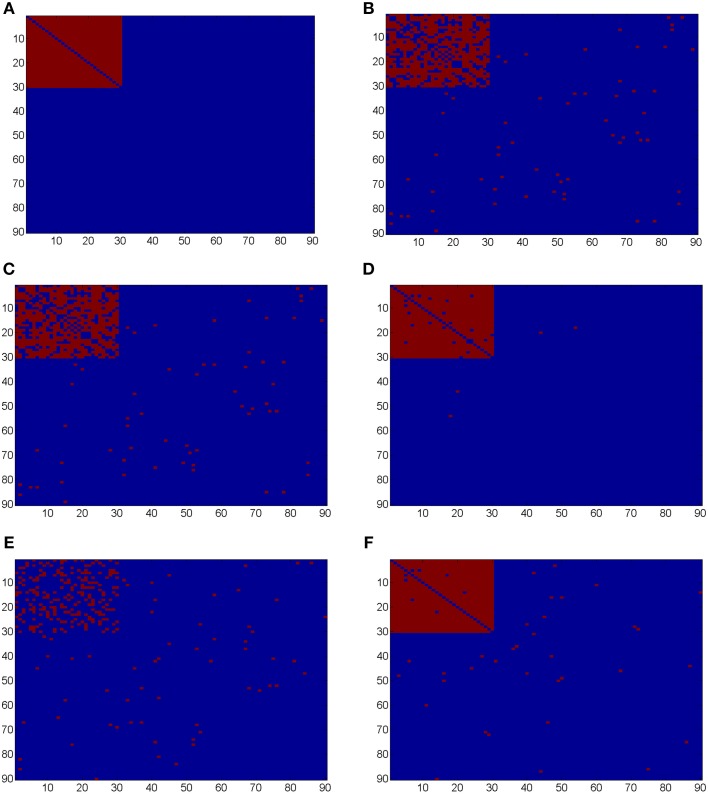
**(A)** Heatmaps of truth the connectivity between the first 30 nodes are differentially expressed between the two groups; **(B–D)** Heatmaps of the test results using Wilcoxon signed-rank test and FDR control with *q* = 0.1 (red = reject and blue = fail to reject) of the original correlations *z*, the probit (variance stablizing) transformed correlation, and the normalized correlations *g*_*s*_(*z*), respectively, under the scenario of no systematic sifts; **(E,F)** Heatmaps of the test results of the original correlations *z* and the normalized correlations *g*_*s*_(*z*) under the scenario of with systematic shifts.

Ideally, the test results reveal the 435 “true positives” with 0 “false positives.” Figure 5 shows the testing results by different methods and scenarios. Figure 5A reflects the true differentially expressed connectivity expressions between the two groups for the first 30 nodes (red) and the rest are at the level (blue). Figure 5B shows the testing results between the two groups based on the non-normalized correlations. Figure 5C are the testing results based on the probit (variance stablizing) transformed correlations (the logit transformation performance is very similar). Figure 5D are the testing results based on the empirical Bayes normalized correlations. Figures 5E,F are the test results of non-normalized and normalized correlations under the scenario with systematic shifts. Based on all the differentially expressed connectivity/biomarker discovery results, the normalized connectivity metrics have much lower type I and II errors. Table 1 summarizes the detailed results with comparison to the truth over 100 times of simulations. The number of false positive testing results of non-normalized correlations is about 17 times of the normalized correlations, and the number of false negative testing results is more than about 20 times; the difference is even larger in the shifted scenario. The performance of probit variance stablizing transformed correlations are similar to the original correlations. The levels of random shift (σ^2^) affect the performance of the differential detection, however after the empirical Bayes normalization the shift almost has no impact on the result findings. Therefore, the simulation study results indicate that our normalization method can effectively scale the connectivity to appropriate level and improves the power to identify the true differentially expressed connectivity with low false positive rate. When the null is more dispersed and connected component is right skewed, the two mixture components are more mixed and thus the false positives and false negatives increase. Yet, our method outperforms the non-normalized correlations for differentially expressed feature detection. Overall, the empirical Bayes normalization model provides a more robust pathway for connectivity expression quantification and enables biomarker discovery with both high sensitivity and specificity.

**Table 1:** **Results of differential expression tests with normalized and unnormalized correlations (without and with systematic shift): mean and standard deviation of 100 simulations**.

**Methods**	**False positives(%)**	**Std**	**False negatives(%)**	**Std**
Correlation	32.4 (0.9)	6.1	127.1 (29.2)	9.6
Probit transformed correlation	34.7 (0.9)	6.0	129.4 (29.7)	10.2
Normalized correlation	2.1 (0.06)	0.3	7.3 (1.7)	1.1
Correlation P^a^_1_	52.9 (1.48)	8.9	186.5 (52.24)	15.4
Normalized correlation P_1_	3.6 (0.1)	0.6	28.1 (6.5)	3.9
Correlation P_2_	68.3 (1.9)	0.6	236.4 (54.3)	31.6
Normalized correlation P_2_	11.8 (0.3)	3.2	43.8 (10.1)	8.2
Correlation P_3_	16.5 (0.4)	4.4	83.5 (19.2)	8.7
Normalized correlation P_3_	1.3 (0.04)	0.3	2.5 (0.7)	0.5
Correlation + shift (σ^2^ = 0.3)	37.1 (1.04)	7.4	306.5 (70.4)	19.5
Normalized correlation + shift (σ^2^ = 0.3)	17.6 (0.9)	2.2	3.5 (0.8)	0.5
Correlation + shift (σ^2^ = 1)	62.3 (1.74)	9.8	326.4 (74.7)	20.1
Normalized correlation + shift (σ^2^ = 1)	19.3 (0.53)	2.9	4.1 (0.9)	0.7

a Please refer to the parameters in paragraph two of the Section 3.

## 4. Data example

This data set was collected at Brain Mapping Center in University of California, Los Angles (UCLA), one of the data collecting sites in the Autism Brain Imaging Data Exchange (ABIDE) (Rudie et al., 2012, 2013; Di Martino et al., 2014). The imaging was performed on Siemens magneto Trio scanners. The imaging data were obtained using a gradient echo T2^*^-weighted echo planar imaging sequence, echo time *TE* = 28 ms, repetition time *TR* = 3 s, 64 × 64 matrix with 34 slices 4.0 mm tick, resulting in whole brain coverage with a voxel size of 3 mm × 3 mm × 4 mm. During the MRI scanning, initially 33 participants (typical controls, TC) and 49 patients with the Autism spectrum disorders (ASD) were asked to lie as still as possible, keep their eyes open, try not to fall asleep, and think about whatever they want. A white background with a black central fixation cross was presented during the resting state scan, although participants were not asked to fixate, they were verified that they had not fallen asleep at the end of the scan. Participants with large motions were removed from the dataset, resulting in 32 participants in the TC group and 41 in the ASD group.

The rs-fMRI data are performed slice time correction and motion correction. The data are registered to a standard MNI space with voxel size 2 mm^3^ and is normalized to be percent signal change. The masks of the white matter (WM), the gray matter (GM), and the cerebrospinal fluid (CSF) are crated in the standard MNI space. The mean time series from the WM and the CSF are calculated. The time series from the GM are regressed out the mean time series of the WM, the CSF and the six movement parameters. A linear trend is removed from all the signal. The fMRI time series are filtered using a bandpass with passing band (0.009–0.08 Hz) and spatially smoothed with 6 mm FWHM Gaussian kernel. We then use the first 90 AAL ROIs as nodes, and take the average of all voxels' temporal profiles within each ROI as region level signal for all subjects (Zalesky et al., 2010b). Four-thousand-five Pearson correlation coefficients are calculated between the 90 nodes, and then Fisher's z transformation are applied. In this analysis, we focus on the differential connectivity expressions between TC and ASD by using normalized connectivity metrics.

**Figure 6:**
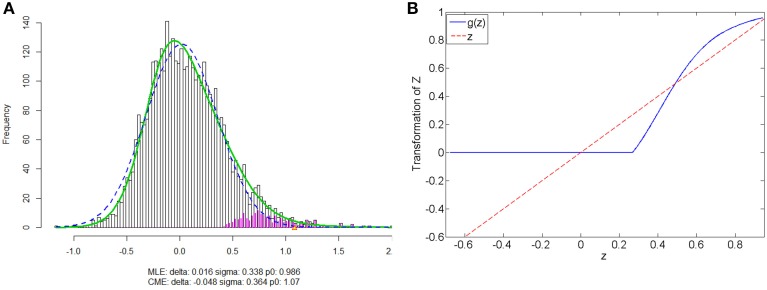
**Subject one in data example: (A) is the mixture model estimation procedure using “*locfdr*” in R for Fisher's z transformed correlation; (B) the normalization function vs. the original correlations: the blue line is the normalization function that maps the raw connectivity metrics to normalized metrics; the red line is used a reference representing no normalization is applied**.

**Figure 7:**
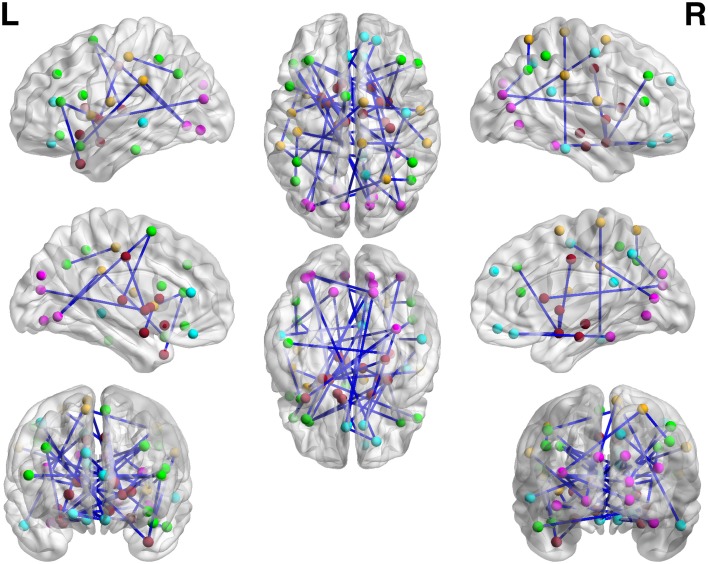
**The regions showed higher correlations in children with ASD, compared to the TD group (*q* < 0.05, corrected for multiple comparisons)**. No pairs of regions showed higher connectivity in the TD than the ASD group.

We apply the normalization algorithm to all 4005 connectivity metrics for each individual, and no subject in this data set is detected with anticorrelation component of the mixture model. Figure 6 shows the distribution of correlations for one subject as well as the corresponding empirical Bayes normalization function. Next, we conduct Wilcoxon signed-rank tests toward all 4005 original correlations and normalized correlations between 90 ROIs for TC vs. TSD. We then perform local fdr for multiple testing control. Unlike the simulation study, the ground truth of the false positives and false negatives of the data example is unknown. Comparing to the simulation testing results, it seems that the difference between test results of original and normalized correlations has the similar pattern: the normalized connectivity test results include small *p*-values scattered randomly. Because 4005 tests are performed simultaneously, the multiple testing correction methods including local *fdr* and Network Based Statistics (NBS) performed for both empirical Bayes normalized correlations and original correlations (Efron, 2004; Zalesky et al., 2010a). No significant feature or network is identified after the correction for the original correlations (*q*-value 0.1 as threshold for local fdr and permutation *p*-value 0.05 for NBS). In contrast, the analysis based on empirical Bayes normalized connectivity metrics shows significant connectivity differences between the ASD and TC groups, and 44 connectivity features have *fdr q*-values less than 0.1. We demonstrate the results in Figure 7. The ASD group show higher function connectivity between pairs of ROIs for all the 44 features than the TC group. Most of these significantly expressed connectivity are between distant ROIs, which are across the the functional subsystems of primary sensory, subcortical, limbic, paralimbic, and association areas defined by Mesulam (1998) and Supekar et al. (2013). We further perform bootstrap analysis to evaluate the reliability of the findings. From 3000 resamples, the 44 features are detected on average 78.6% (with sd 11.3%). As comparison, we detect no connectivity between or within any of these subsystems showing greater connectivity in the TD group, compared with the ASD group. These results suggest that hyper-connectivity in ASD spans multiple functional subsystems of the human brain. The revealed results are consistent with the recent findings of brain hyper-connectivity of ASD children by Supekar et al. (2013), which include multiple studies from three image data acquisition sites in the U.S.

We note that the results can only be identified by using the empirical Bayes normalized connectivity metrics, but not by the original connectivity metrics. Therefore, the normalization step is essential for rs-fMRI based brain connectivity study, and our empirical Bayes normalization method provides a sound pathway to successfully fulfill the task.

## 5. Discussion

In this article, we have presented a novel empirical Bayes method for rs-fMRI connectivity metric normalization, and the simulation study and the data example have shown that the quantification and statistical inferences based on the normalized inputs are more powerful and reliable. The normalization step has been widely used in high-throughput biomedical data analysis with the goal to remove systematic measurement error generated in the complex data acquisition and preprocessing steps and to improve the validity and reproducibility of the following statistical analyses. It has been discussed that a preprocessing step of global signal regression could shift the distributions of the correlations and influence the statistical inferences (Fox et al., 2009; Murphy et al., 2009; Weissenbacher et al., 2009). There may be many other latent factors to affect the quantification of the connectivity metrics as well. Therefore, we feel that normalization toward connectivity metrics should be introduced.

### 5.1. Quantification of brain functional connectivity metrics

Different from the high-throughput “omics” data, the brain functional connectivity is not measured directly but rather calculated by some statistics/metrics based on a pair of time courses from fMRI data. It is unclear how the calculated statistics/metrics can appropriately reflect the true connectivity strength and are comparable across subjects, regardless what statistic is chosen (e.g., correlation coefficient or mutual information coefficient). It is possible to obtain extremely large absolute value correlations between two white noise vectors, which gives rise to the false positive discovery. From the statistical perspective, most connectivity statistics can be proved to follow a known distribution asymptoticly and accordingly the *p*-values are calculated with both type I and II errors. Comparing with the conventional normalization method such as quantile normalization, the empirical Bayes mixture model lends itself to incorporating the false positive concept into quantification of the functional connectivity expression and provides a (posterior) probability based scale. The data driven (rather than a deterministic linear/nonlinear transformation) quantification method could provide a more comparable scale for group level connectivity inferences. For example, a 0.1 difference in original correlations could be mapped to around 0.5 difference in the normalized correlations at the interaction between two components due to the increase of posterior probability of true positive. The amplified difference tend to improve the subtle difference detection, because it can better represent connectivity strength. The computational techniques for the mixture model estimation have been developed for local *fdr* estimation by Efron (2004) and Wu et al. (2006), which provides us a convenient tool to calculate the subject-specific normalization function. The only assumption of our method is that the majority (*p*_0_ > 0.9) of connectivity expressions are from the null distribution, which needs to further verified with more rs-fMRI studies. The assumption is generally valid, and all connectivity metric distributions of the data sets we tested follow such pattern. If the assumption is violated, Wu et al. (2006) provides promising numerical solution using nonparametric curve fitting methods. Moreover, another obvious advantage of the normalization method is that it maps the correlations to the range of [0, 1] by the empirical Bayes posterior probability normalization function, which avoids the information loss due to hard thresholding of correlations in complex network analysis using graph theoretical models (Rubinov and Sporns, 2011).

The appropriate brain connectivity metric normalization method improves the power to detect the truly differentially expressed features and yield less false positive findings. In the simulation study, we compare the test results based on different connectivity metrics with reference to ground truth, and it shows the empirical Bayes normalized correlation has the lowest type I and II errors and is more robust to systematic shifts. When applying our method to the data example, the analysis results based on normalized connectivity metrics detect hyper-connectivity between pairs of regions from distant functional subsystems for the ASD group with comparing to TC group. Such features are not detected by using the non-normalized correlations. The findings align with the results by Supekar et al. (2013) which performs between region connectivity analysis for several autism studies from different sites. Supekar et al. (2013) also provides explanation of these findings from the perspectives of neuroscience and the link to clinical symptoms of ASD. The practical brain connectivity study using neuroimaging technology often involves multiple steps of numerical analysis which are subject to many unavoidable errors and noises, and we feel that the empirical Bayes normalization improves both power and reliability of statistical analysis.

### 5.2. Anticorrelations

**Figure 8:**
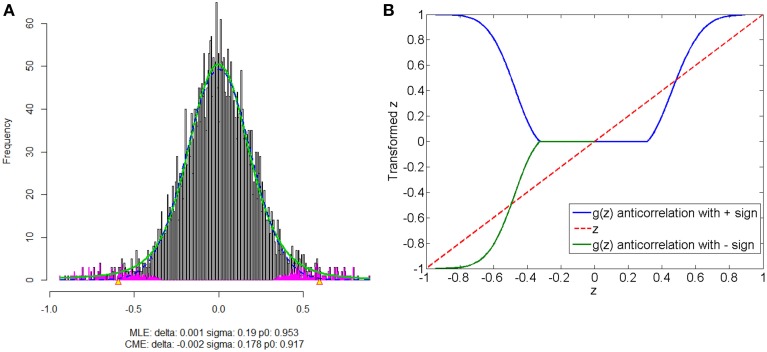
**Connectivity metrics with both correlated and anticorrelated components: (A) is the mixture model estimation procedure using “*locfdr*” of the three components; (B) the original correlations vs. the normalized correlations: the blue line is the posterior probability of the correlated component and anticorrelated component are >0 (“+” sign), and the green line discriminate correlated or anticorrelated posterior probability by using a “−” sign to indicate whether it anticorrelated**.

The anticorrelations in rs-fMRI data have drawn attention of many neuroimaging researchers (Fox et al., 2009; Murphy et al., 2009; Weissenbacher et al., 2009; Chai et al., 2012). The discussion has not reached to the agreement whether the anticorrelations are “true positive” or “false positive.” The proposed normalization method provides a pathway to automatically detect the “true positive” anticorrelation component by classifying the “true positives” and “false positives” based on the empirical distribution of connectivity metric. Figure 8 shows that correlated and anticorrelated components can be identified, if existing, we could assign either “+” or “−” sign to anticorrelated connectivity metric depending on different following analyses. Generally, “−” sign suits the regression analysis or statistical tests better, because anticorrelation could be considered as the opposite of correlation. When applying the graph theoretical model based network analysis using normalized connectivity, two separate analyses should be conducted for correlations and anticorrelations (with “+” sign) if both components are detected, with the normalized connectivity metric range of [0, 1] (in Figure 8B). Thus, the results include two parts of inferences: properties of correlated networks and anticorrelated networks. Although in our data example there is no anticorrelation component detected, that normalization method can be also applied to deal with anticorrelations in practical data analysis. Yet, out normalization method could be combined with pre-processing steps (e.g., global signal regression), as the normalized connectivity is probability and shift-invariant.

## 6. Conclusion

In summary, a new rs-fMRI connectivity metric normalization method has been developed and applied to functional brain connectivity analysis. The better connectivity normalization/quantification methods yield generally higher reproducibility. Although we utilize the Pearson correlation coefficient as connectivity metric and rs-fMRI for demonstration, we are optimistic that the developed method are ready to be applied to the task-induced fMRI connectivity study and other connectivity metrics because the empirical Bayes framework is flexible to fit various distributions of connectivity metrics.

### Conflict of interest statement

The authors declare that the research was conducted in the absence of any commercial or financial relationships that could be construed as a potential conflict of interest.
